# The Risk Threshold for Hemoglobin A1c Associated With Albuminuria: A Population-Based Study in China

**DOI:** 10.3389/fendo.2021.673976

**Published:** 2021-05-31

**Authors:** Hong Lian, Hongshi Wu, Jie Ning, Diaozhu Lin, Chulin Huang, Feng Li, Ying Liang, Yiqin Qi, Meng Ren, Li Yan, Lili You, Mingtong Xu

**Affiliations:** ^1^ Department of Endocrinology, Sun Yat-sen Memorial Hospital, Sun Yat-sen University, Guangzhou, China; ^2^ Department of Metabolic Endocrinology, Shenzhen Longhua, District Central Hospital, Shenzhen, China

**Keywords:** HbA1c, albumin to creatinine ratio, albuminuria, diabetic kidney disease, cross-sectional study

## Abstract

**Background:**

Diabetic kidney disease (DKD) is a kind of common microvascular complication of diabetes. This study aims to explore the possible links between blood sugar level and albuminuria, providing the exact cut point of the “risk threshold” for blood glucose with DKD.

**Methods:**

The relationship between blood glucose and albuminuria was modeled using linear and logistic regression in the REACTION study cohorts (N= 8932). Odds ratios (ORs) and 95% confidence intervals (CIs) were calculated by logistic regression model. Two-slope linear regression was used to simulate associations between blood glucose and ACR.

**Results:**

We found that the increase in ACR was accompanied by increased HbA1c, with a turning point at 5.5%. The positive correlation remained highly significant (P<0.001) when adjusted for age, sex, marital status, education, smoking status, drinking status, BMI, waistline, SBP and DBP. In subgroup analyses including gender, obesity, hypertension, and smoking habits, the relationship was significant and stable.

**Conclusions:**

We determined a risk threshold for HbA1c associated with albuminuria in a Chinese population over the age of 40. HbA1c ≥ 5.5% was positively and independently associated with ACR. These results suggest the necessity of early blood glucose control and renal function screening for DKD in at-risk populations.

## Introduction

Diabetic kidney disease (DKD) is the most common microvascular complication of diabetes and the most common cause of end-stage renal disease (ESRD) (1). About 30-40% of people with diabetes develop DKD and half of the patients with DKD progress to ESRD (2), adding additional mortality and healthcare costs to patients (3, 4). Albuminuria is a marker of renal/glomerular disease and a key indicator of DKD (5). Albuminuria is a pathophysiological event triggered by several factors, including: damage to the endothelial glycocalyx, loss of endothelial cell function and rearrangement and injury of the cytoskeleton of podocytes (6). Accumulation of glucose and glycosylated proteins in endothelial cells leads to impaired glomerular filtration barrier function and changes in glomerular capillary permeability in patients with diabetic nephropathy (7, 8). Urinary albumin measurements are typically used to identify and monitor patients with kidney dysfunction (9) by measuring the ratio of albumin to creatinine (ACR) in urine (10, 11). Many studies have confirmed that high ACR indicates glomerular ultrafiltration and is related to renal failure, acute kidney injury, and progression of renal disease (12–14). An increase in urinary albumin excretion is widely recognized as a precursor of diabetic kidney disease, predicting an increased early risk and suggesting the need for early clinical intervention (15).

Hemoglobin A1c (HbA1c) represents the average fluctuation level of blood glucose in the body in recent three months. As a significant change in clinical practice, the American Diabetes Association’s 2010 guidelines recommended HbA1c as one of the diagnostic criteria for diabetes, and set a threshold of ≥ 6.5% (16). Since the DCCT (Diabetes Control and Complications Trial) and UKPDS (UK Prospective Diabetes Study) (17) showed that HbA1c level is a strong predictor of the risk of diabetic microvascular complications, glycosylated hemoglobin level has been the focus of diabetes management. Intensive glycemic therapy has been shown to reduce the incidence of albuminuria and delayed DKD progression to some extent (18). Currently, many studies have confirmed that HbA1c>7.0% is significantly associated with ACR and served as a risky indicator for severe DKD (19–21). However, the risk threshold of glycosylated hemoglobin observed in these studies had no obvious inflection point. Furthermore, there is evidence that the onset of microvascular complications may occur prior to the diagnosis of diabetes, so the above experiments may not accurately reflect the exact point at which the risk of DKD begins to increase.

The objective of our research is to explore the correlation between blood glucose and the continuous increase in ACR values and to reveal the adverse effects of hyperglycemia on renal function prior to the diagnostic criteria for diabetes being met. We attempt to determine the optimal blood glucose threshold to provide additional clinical evidence for the prevention and early screening of diabetic kidney injury, so as to prevent the occurrence and development of DKD.

## Materials and Methods

### Study Population

We conducted a cross-sectional study of community populations from March to June 2011. Our research population was obtained from the longitudinal REACTION study, which aimed to assess cancer risk and related risk factors in Chinese diabetic population. Specific information regarding experimental design and cohort recruitment has been previously reported (22, 23). Originally, we recruited a total of 10,104 residents over the age of 40, while 188 subjects did not agree to participate in the baseline investigation and were excluded from the analyses, yielding a participation rate of 98.1%. Subjects with missing information (waistline, n=222; body mass index (BMI), n=330; fasting plasma glucose (FPG), n=201; oral glucose tolerance test for 2 hours (OGTT 2 h), n=249; hemoglobin A1c (HbA1c), n=235; albumin-to-creatinine ratio (ACR), n=135; and estimated glomerular filtration rate (eGFR), n=16) were excluded from analyses. Finally, 8,932 subjects were included in the analysis (**Supplementary Figure 1**).

### Clinical and Biochemical Indicators

We designed a questionnaire survey to gather information on demographic data (marital status, employment and educational level) and living habits (smoking and drinking). Education level was categorized as elementary school or below, junior high school, technical secondary or high school, and college degree or above. Smoking and drinking habits were defined as never, former, or current, indicating who had regularly smoked or consumed alcohol during the past 6 months.

Participants received a physical examination. Their height and weight were measured in units of 0.1cm and 0.1kg, and BMI (kg/m2) was calculated. Waistline was defined as the horizontal circumference through the umbilical center measured with a soft tape measure, at the end of exhalation and before the beginning of inhalation. Hipline was measured at the most convex region of the pubic symphysis and the gluteus maximus, with the legs close together and the arms resting naturally at the sides. Waist-to-hip ratio (WHR) was calculated as waistline divided by Hipline. Blood pressure was measured after at least 5 minutes of rest, and the average of three measurements was taken (OMRON, Omron Company, Dalian, China).

After overnight fasting for at least 8 hours, we collected venous blood for laboratory tests. FPG and serum creatinine were measured by an autoanalyzer (Beckman CX-7 Biochemical Autoanalyzer, Brea, CA, USA). The level of HbA1c was detected by high-performance liquid chromatography (Bio-Rad, Hercules, CA). For the OGTT test, blood glucose levels were measured 0h before and 2h after 75g glucose orally. We collected the first morning urine sample of participants for laboratory testing. Urinary albumin levels in the samples were determined by chemiluminescence immunoassay (Siemens Immulite 2000, USA). And creatinine levels were determined using Jaffe kinetic method (Biobase-Crystal, Jinan, China), respectively. ACR was estimated as the urine albumin to creatinine ratio and is expressed as mg/g. eGFR took mL/min per 1.73 m^2^ as the unit and used the following formula: eGFR = 175 × [serum creatinine × 0.011]^-1.234^ × [age]^-0.179^ × [0.79 if female] (24).

### Definition of Diabetes, CKD, Hypertension, and Central Obesity

The diagnostic criteria for diabetes are based on the 1999 World Health Organization (WHO) criteria, including fasting blood glucose greater than or equal to 7.0 and/or OGTT 2 h greater than or equal to 11.1, or self-reported history of diabetes (25). We included systolic blood pressure ≥140 mmHg and/or diastolic blood pressure ≥90 mmHg or participants reported a previous history of hypertension as patient with hypertension (26). Definition of central obesity is a waistline ≥ 90 cm in men and ≥ 80 cm in women (27). The criteria of chronic kidney disease (CKD) is eGFR less than 60 mL/min per 1.73 m^2^ or the presence of persistent severely elevated albuminuria (an albumin-to-creatinine ratio (ACR) of >30 mg/g) (28).

### Statistical Analysis

Continuous variables that satisfy a normal distribution are presented as the mean ± standard deviation (SD), one-way ANOVA was used to analyze group differences, and Bonferroni correction was performed for *post hoc* comparisons. Continuous variables with nonnormally distributed data are indicated as median and interquartile range (IQR), and Kruskal-Wallis test was performed to compare group differences. Categorical variables are presented as numbers (proportions), and differences between groups were performed with the χ2 test. The unadjusted and multivariate-adjusted linear and logistic regression analysis, calculating odds ratios (ORs) and corresponding 95% confidence intervals (95% CI), were used to identify risk factors for increased risk for diabetes. A 2-slope linear regression was used to model associations between HbA1c and ACR. Adjustment was then made *a priori* for relevant covariate: age, sex, marital status, education, smoking status, drinking status, BMI, waistline, systolic blood pressure, diastolic blood pressure, fasting plasma glucose, oral glucose tolerance test and HbA1c. To determine whether eGFR CKD, hypertension and central obesity interact with HbA1c increments in predicting ACR, product terms for (eGFR ≥ 60) × HbA1c, CKD × HbA1c, hypertension × HbA1c, central obesity × HbA1c were added into the regression model with covariate adjustments as mentioned above. All statistical analyses were performed using RStudio version 3.6.1. A two tailed p<0.05 was considered to be statistically significant.

### Patient and Public Involvement Statement

This research finished without patient involvement. Patients were not invited to participate in research design, data analysis and manuscript writing.

## Results

### Basic Characteristics of the Study Population

A total of 8,932 participants completed the collection of demographic, physical examination, and laboratory data. Clinical characteristics of the participants are shown according to ACR category grouped by quartile in **Table 1**. Higher ACR was accompanied by increased age, BMI, waistline, Hipline, WHR, SBP, FPG, OGTT 2-h glucose, and HbA1c, as well as reduced DBP, height, and current smoking (all p for trend <0.001).

**Table 1:** Characteristics of participants by ACR category.

Variables	ACR Category, mg/g				
	Q1 (≤5.93)	Q2 (5.94-7.80)	Q3 (7.81-11.70)	Q4 (≥11.70)	P_trend_
Sample, n	2232	2234	2232	2233	–
Age, years, mean ± sd	54.74 ± 7.36	55.61 ± 7.50**	55.83 ± 7.48**	56.46 ± 8.13**	<0.001
Male, n%	885 (39.65)	644 (28.83)**	481 (21.55)**	491 (21.99)**	<0.001
Married or Cohabitation, n%	2054 (92.48)	2022 (91.16)	2007 (90.49)	1950 (87.84)**	<0.001
Education, n%					
Elementary school or below	178 (8.24)	267 (12.20)**	274 (12.50)**	300 (13.80)**	<0.001
Junior high school	548 (25.37)	582 (26.60)	594 (27.11)	636 (29.25)*	0.003
Technical secondary or high school	1163 (53.84)	1140 (52.10)	1149 (52.44)	1071 (49.26)*	0.011
College degree or above	271 (12.55)	199 (9.10)**	174 (7.94)**	167 (7.68)**	<0.001
Heigth, cm, mean ± sd	160.0 ± 7.6	158.7 ± 7.3**	157.4 ± 7.2**	157.2 ± 7.1**	<0.001
Weight, kg, mean ± sd	59.9 ± 9.0	58.7 ± 9.1**	57.8 ± 8.9**	59.4 ± 9.6	0.003
BMI, kg/m^2^, mean ± sd	23.36 ± 2.82	23.27 ± 2.94	23.30 ± 2.90	23.98 ± 3.18**	<0.001
Waistline, cn, mean ± sd	81.01 ± 8.44	80.89 ± 8.88	80.70 ± 8.67	82.37 ± 9.28**	<0.001
Hipline, cm, mean ± sd	93.59 ± 6.15	93.53 ± 6.57	93.53 ± 6.46	94.42 ± 6.78**	<0.001
WHR, mean ± sd	0.87 ± 0.06	0.86 ± 0.06	0.86 ± 0.06	0.87 ± 0.07*	0.009
Current smoking, n%	285 (12.96)	213 (9.72)*	190 (8.67)**	173 (7.87)**	<0.001
Current drinking, n%	75 (3.42)	75 (3.41)	75 (3.42)	63 (2.87)	0.334
SBP, mmHg, mean ± sd	122.7 ± 14.5	124.6 ± 15.7*	124.5 ± 14.9**	128.8 ± 16.2**	<0.001
DBP, mmHg, mean ± sd	74.1 ± 9.2	75.0 ± 9.3*	74.6 ± 9.2	76.4 ± 10.1**	<0.001
Hypertension, n%	465 (21.78)	576 (26.74)**	605 (28.02)**	868 (40.26)**	<0.001
FPG, mmol/L, mean ± sd	5.46 ± 0.78	5.49 ± 0.78	5.52 ± 0.79	5.58 ± 0.93**	<0.001
OGTT 2h glucose, mmol/L, mean ± sd	7.46 ± 2.24	7.69 ± 2.40*	7.76 ± 2.46**	8.13 ± 2.23**	<0.001
HbA1c, %, mean ± sd	5.18 ± 0.49	5.91 ± 0.52**	5.97 ± 0.51**	6.02 ± 0.60**	<0.001
Diabetes, n%	215 (9.73)	280 (12.66)*	313 (14.21)**	425 (19.26)**	<0.001

Data were means ± SD or medians (interquartile ranges) for skewed variables or numbers (proportions) for categorical variables.

P for trend was calculated for the linear regression analysis tests across the groups.

P values were for the ANOVA or χ ^2^ analyses across the groups.

*P<0.05, **P<0.001 compared with quantile 1 (ACR ≤ 5.93 mg/m).

BMI, body mass index; SBP, systolic blood pressure; DBP, diastolic blood pressure; FPG, fasting plasma glucose; OGTT, oral glucose tolerance test; ACR, albumin:creatinine ratio.

### The Relationship Between Glucose and ACR

**Figure 1:**
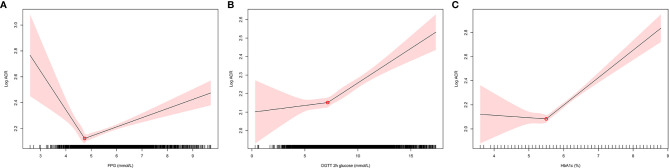
The relationships between blood glucose and log-ACR were examined using 2-slope linear regression models. The inflection points of log-ACR with **(A)**: FPG, **(B)**: OGTT 2h glucose, **(C)**: HbA1c. FPG, fasting plasma glucose; OGTT, oral glucose tolerance test; ACR, albumin:creatinine ratio.

ACR was positively skewed and was logarithmically transformed before linear regression analysis. The relationships between blood glucose and log-ACR were examined using 2-slope linear regression models. As shown in **Figure 1**, the inflection points occurred at 4.74 mmol/L for FPG, 7.16 mmol/L for OGTT 2-h glucose and 5.5% for HbA1c.

We next explored the association between glucose levels and ACR in continuous using linear regression analyses, as well as ACR ≥ 30 in category using logistic regression analysis in **Table 2**. FBG ≥ 4.74 mmol/L and OGTT 2-h glucose ≥ 7.16 mmol/L were accompanied by higher ACR in the unadjusted model. However, in the adjusted model, which was adjusted for age, sex, marital status, education, smoking status, drinking status, BMI, waistline, SBP and DBP, the positive correlation disappeared. Surprisingly, both in unadjusted and adjusted models of linear regression and logistic regression, HbA1c ≥ 5.5% remained significantly (P<0.001) positively associated with ACR.

**Table 2:** Regression models of the relationship between glucose and ACR.

Glucose category	Fold-Change per 1 unit increase in glucose component (95%CI)			
	ACR (mg/g)^a^		ACR ≥30 (mg/g)^b^	
Unadjusted model				
FPG<4.74 mmol/L	0.752 (0.627-0.901)	P=0.021	0.677 (0.314-1.621)	P=0.349
FPG≥4.74 mmol/L	1.074 (1.050-1.099)	P<0.001	1.308 (1.172-1.454)	P<0.001
OGTT 2h <7.16 mmol/L	1.021 (0.991-1.052)	P=0.166	1.174 (1.029-1.328)	P=0.013
OGTT 2h ≥7.16 mmol/L	1.033 (1.026-1.040)	P<0.001	1.145 (1.106-1.184)	P<0.001
HbA1c <5.5%	0.981 (0.839-1.146)	P=0.807	0.741 (0.329-1.876)	P=0.496
HbA1c ≥ 5.5%	1.256 (1.210-1.305)	P<0.001	2.033 (1.723-2.387)	P<0.001
Adjusted model^#^				
FPG<4.74 mmol/L	0.743 (0.613-0.900)	P=0.003	0.711 (0.308-1.842)	P=0.451
FPG≥4.74 mmol/L	0.978 (0.950-1.006)	P=0.134	0.887 (0.757-1.035)	P=0.132
OGTT 2h <7.16 mmol/L	0.995 (0.964-1.027)	P=0.756	1.046 (0.894-1.211)	P=0.563
OGTT 2h ≥7.16 mmol/L	1.007 (0.999-1.016)	P=0.092	1.070 (1.020-1.122)	P=0.006
HbA1c <5.5%	0.896 (0.761-1.054)	P=0.185	0.642 (0.280-1.659)	P=0.325
HbA1c ≥ 5.5%	1.256 (1.197-1.318)	P<0.001	1.808 (1.422-2.294)	P<0.001

FPG, fasting plasma glucose; OGTT, oral glucose tolerance test; ACR, albumin:creatinine ratio.

a linear regression models for log of ACR (mg/g);

b logistic regression models for ACR ≥30 (mg/g);

^#^Covariates in the adjusted model: age, sex, status of marriage, education, smoking status, drinking status, BMI, waistline, systolic blood pressure, diastolic blood pressure, FPG, OGTT 2h and HbA1c.

### Relationship Between HbA1c and ACR

To examine the independent effects of eGFR, CKD, hypertension, central obesity on the relationship between HbA1c and ACR, interaction terms were introduced into the linear regression model. Back-transformed coefficients from this model are presented in **Table 3**. In the whole population, one unit increase in HbA1c was associated with an adjusted increase in ACR of 1.164-fold. There was also significant interaction between HbA1c and ACR: each unit increase in HbA1c was accompanied by a further adjusted increase in ACR of 1.161, 1.133, 1.143 and 1.161 for participants with eGFR≥60, CKD, hypertension, central obesity, respectively (**Table 3**).

**Table 3:** Interactions between CKD, hypertension, HbA1c and ACR.

HbA1c term	Fold change (95%CI) of log ACR for per unit increase in HbA1c	
Whole population coefficient	OR (95%CI): 1.164 (1.121-1.208)	P<0.001
Further increment from interactions		
(eGFR ≥ 60) × HbA1c	OR (95%CI): 1.161 (1.118-1.205)	P<0.001
CKD × HbA1c	OR (95%CI): 1.133 (1.100-1.167)	P<0.001
Hypertension × HbA1c	OR (95%CI): 1.143 (1.093-1.194)	P<0.001
Central obesity × HbA1c	OR (95%CI): 1.161 (1.106-1.219)	P<0.001

ACR, albumin:creatinine ratio; eGFR, estimated glomerular filtration rate; CKD, chronic kidney disease.

Model adjusted for age, sex, status of marriage, education, smoking status, drinking status, BMI, waistline, systolic blood pressure, diastolic blood pressure, fasting plasma glucose, oral glucose tolerance test and HbA1c.

**Figure 2:**
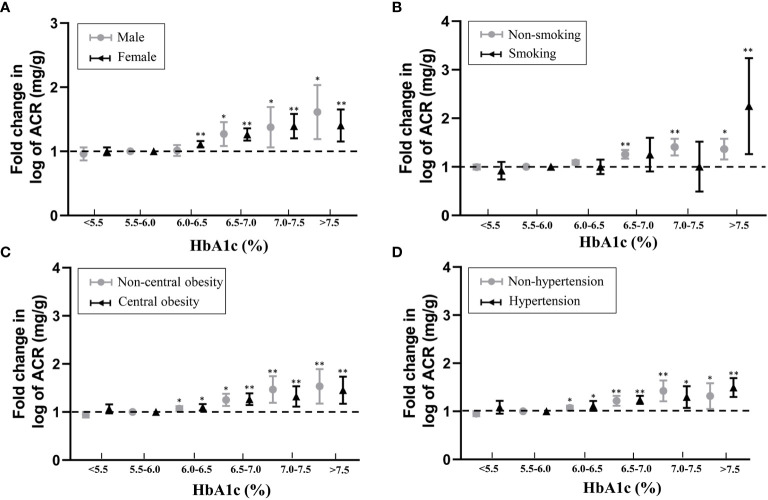
Associations between HbA1c and fold-change in log-ACR in subgroup analyses taking into account with **(A)** gender, **(B)** smoking habits, **(C)** obesity and **(D)** hypertension. Fold change is relative to 5.5% ≤ HbA1c<6.0% referent category. *P < 0.05, **P < 0.001.

The subgroup analyses took into account gender, obesity, hypertension, and smoking habits, and the positive relationship between HbA1c and ACR remained stable and significant. This positive correlation was not affected by central obesity, hypertension or gender (**Figure 2**).

## Discussion

Our study provides evidence that among blood glucose indicators, only HbA1c is independently associated with ACR. The risk threshold identified for HbA1c for the prevalence of albuminuria among a Chinese population was 5.5%. The increase in ACR was accompanied by a positive change in HbA1c, when HbA1c ≥ 5.5%. Furthermore, the correlation remained stable and significant after adjusting for sociodemographic characteristics and performing subgroup analyses based on gender, smoking habit, obesity, and hypertension. This result indicates the effect of blood sugar on renal damage may occur before the diagnosis of diabetes, emphasizing the necessity of early blood glucose control and renal function screening for DKD.

At present, many studies have shown that HbA1c levels affect albuminuria. For people with diabetes, data from the FinnDiane study and DMIDS project both suggest that HbA1c influences the development of albuminuria and renal disease (29, 30). Meanwhile, the AdDIT study, which was focused on adolescents, reached the same conclusion (31). The above results are consistent with those of our study. However, these analyses were performed only in a diabetic population with HbA1c >7% among enrolled subjects and showed a linear relationship without obvious inflection points. In addition, prediabetes is also an independent risk factor for glomerular ultrafiltration and increased ACR. The NHANES and the ARIC studies demonstrated that microvascular dysfunction is already present in prediabetes, and there was a significant trend toward an increased risk of kidney disease and ESRD with increased baseline HbA1c levels (32–34). In our analysis, we included the entire study population with levels of HbA1c ranging from 4% to greater than 9%. This result indicates that ACR accompanied by increased HbA1c reaches a turning point at 5.5%, suggesting this as the HbA1c threshold for developing kidney dysfunction.

With respect to a glucose threshold in terms of HbA1c levels, current clinical studies have not consistently demonstrated a relationship for predicting the onset of DKD. In the diabetic population, the ADVANCE study showed no significant increase in the risk of retinopathy or renal complications with an HbA1c level below 6.5% (48 mmol/mol) (35). This result was indistinguishable from diagnostic criteria and only included for those with confirmed diabetes. The ESTHER study demonstrated that the risk for reduced kidney function with respect to eGFR linearly increased to more than three-fold when HbA1c levels were increased, with a turning point at HbA1c = 6.4% (36). In our study, the inflection point is the effect corresponding to ACR, speculating that the increase of ACR occurs earlier than the decline of eGFR in DKD (37, 38). Normal glomerular filtration barriers include porous glomerular endothelium, glomerular basement membrane and podocyte foot processes. For pathological reasons, endothelial dysfunction is the key factors for the damage of filtration membrane during the progression of DKD (38, 39). Chronic hyperglycemia induces oxidative stress that impairs endothelial function and podocytes, leading to impaired glomerular filtration barrier, glomerular hyperfiltration, and increased albumin excretion. In both type 1 and type 2 diabetes patients, a triad of endothelial cell glycocalyx damage, increased vascular permeability and albuminuria occurred (40, 41). The ORIGIN trial followed type 2 diabetes or prediabetes mellitus patients for 6.2 years, and findings suggested a gradual increase in the risk of adverse renal outcomes, from the lowest group (HbA1c< 5.7%) to the highest group (HbA1c > 7.4%) (42). However, the analytic method of that study divided HbA1c into five groups based on the hazard ratio, so only a range in blood glucose threshold was given. Evidence of continuous changes in blood sugar was lacking, and there was no precise inflection point. In our study, we identified an important cutoff at 5.5%, and to our surprise, it did not meet the diagnostic criteria for prediabetes, which is defined as HbA1c of 5.7–6.4% (39–47 mmol/mol) according to the 2019 ADA clinical standards (16). The classification of pre-diabetes diagnostic criteria is still controversial. Many studies have suggested the use of different HbA1c cut-off values to diagnose pre-diabetes for different populations due to genetic and biological differences (43–45). Our result places particular emphasis on people with HbA1c greater than 5.5% potentially having an increased risk for albuminuria and provides more clinical evidence for the diagnosis of prediabetes in Chinese population.

Currently, there is sufficient evidence that intensive glucose therapy significantly reduces albuminuria and improves the composite end point of nephropathy. As of 2019, the ADA recommended target for T2DM glycemic control is HbA1c < 6.5% after adequate consideration of the risk of hypoglycemia. The DCCT study of a T1DM population, the UKPDS study of a T2DM population, and the ADVANCE study all showed that intensive glycemic therapy significantly improved renal clinical benefits compared to conventional glycemic control (46, 47). These studies only focused on people who already had diabetes, the glycemic control threshold and risk of proteinuria were different from those of the general population. There is a lack of recommendations for glycemic control in the normal population who did not meet the diagnostic criteria for diabetes to avoid diabetic kidney injury. Our results provide strong evidence for predicting the starting point of renal function impairment and the glycemic control threshold. With the development of treatments and the popularization of physical examination, current strategies for managing blood glucose should also consider the risk of disease in potentially at-risk populations and evaluate the clinical benefits of early intervention.

The National Institute for Clinical Excellence (UK) and the Standards of Medical Care in Diabetes from ADA both recommend kidney care for all people with type 2 diabetes that includes measuring ACR or albumin levels annually (48, 49). Based on the results of our study, we recommend early glycemic management and kidney function screening of ACR levels for people with risk factors. In terms of the duration of DKD, the timing of intervention may be critical (50). Early and more focused interventions for at-risk populations have the potential to convey substantial, long-term improvements in public health. Therefore, it is particularly important that public health and health care policies place greater emphasis on achieving early glycemic control and complication screening in at-risk populations of DKD (51–53).

Our analysis had the following limitations. First, the design of this study was a cross-sectional survey, which can only identify the correlation but cannot conclude cause and effect between ACR and HbA1c. Second, although we used two regression models to estimate a single point of change, the choice of the point of change may also be skewed. However, this deviation, while having a slight effect on the regression coefficient, does not affect the nonlinear conclusion. Furthermore, while we adjusted for many of the covariables associated with ACR in the multiple regression analysis, other potential factors, such as social status, personal income level, and family lifestyle may also influence the regression results and should be included in the adjustment. Third, we only used morning urine samples to measure ACR levels, but levels of ACR fluctuate with time. Therefore, combining this measurement with 24-hour urine albumin quantitative results to assess the risk of albuminuria would be more scientific and rigorous. However, there was a good correlation between on-site urine ACR samples and urine samples collected 24 hours a day. Urine ACR assessment through field samples may be a reliable alternative to epidemiological specimen collection (54). Fourth, the population in this study primarily included community residents over 40 years old of Chinese subjects. Therefore, the current findings cannot be reliably extrapolated to other races or age groups. Fifth, our analysis lacked relevant information on lowering glucose treatment for diabetic patients and did not analyze the effect of lowering glucose treatment in the model for correction. However, studies have found that HbA1c appears to be an independent risk factor for the development of albuminuria even adjusted for use of antidiabetic drugs (55, 56). Therefore, we suggested that the missing information on glucose-lowering therapy in diabetic patients may not influence the relationship between HbA1c and ACR. However, the study will be more rigorous if detailed information of antidiabetic therapies could be gathered.

## Conclusions

Our study provides evidence for an HbA1c threshold in which the risk of albuminuria begins to increase at a cutoff point of 5.5% in a Chinese population. We found that HbA1c≥5.5% was positively and independently associated with ACR. After adjusting for the influence of risk factors, the correlation remained stable and significant. This finding underlines the need for screening renal function in at-risk populations and the importance of early glucose management to delay the development of renal complications from diabetes.

## Data Availability Statement

Data are available upon reasonable request. Main document data and additional unpublished data from the study are available by sending Email to xumt@mail.sysu.edu.cn with proper purposes.

## Ethics Statement

The studies involving human participants were reviewed and approved by Ruijin Hospital Ethics Committee Shanghai JiaoTong University School of Medicine. The patients/participants provided their written informed consent to participate in this study.

## Author Contributions

All authors contributed to the conception of the study, execution of data collection, data analysis or writing and revision of the paper. All authors contributed to the article and approved the submitted version.

## Funding

This work was supported by National Key R & D Program of China (No. 2016YFC0901204), Medical Scientific Research Foundation of Guangdong Province of China (A2019040), and National Natural Science Foundation of Guangdong, China (2019A1515011110).

## Conflict of Interest

The authors declare that the research was conducted in the absence of any commercial or financial relationships that could be construed as a potential conflict of interest.

## Supplementary Material

Supplementary Figure 1 Flowchart of the study participants selection from the REACTION study. BMI, body mass index; FPG, fasting plasma glucose; OGTT, oral glucose tolerance test; ACR, albumin:creatinine ratio; eGFR, estimated glomerular filtration rate.

## Abbreviations

ACR, albumin:creatinine ratio; DKD, diabetic kidney disease; FPG, fasting plasma glucose; OGTT, oral glucose tolerance test; eGFR, estimated glomerular filtration rate; BMI, body mass index; WHR: Waist-to-hip ratio; SBP, systolic blood pressure; DBP, diastolic blood pressure.
