# Supplementary figures and images for: The Risk Threshold for Hemoglobin A1c Associated With Albuminuria: A Population-Based Study in China
Source: Front Endocrinol (Lausanne). 2021 May 31;12:673976. doi: 10.3389/fendo.2021.673976 (PMC8202121; doi:10.3389/fendo.2021.673976)

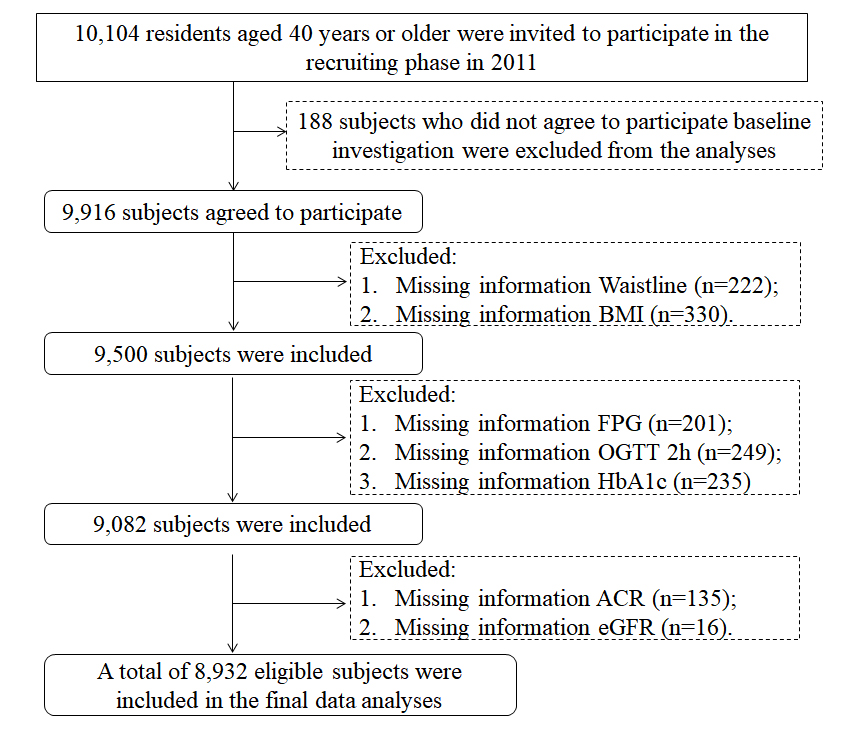

Supplement: Supplementary Figure 1 — Flowchart of the study participants selection from the REACTION study. BMI, body mass index; FPG, fasting plasma glucose; OGTT, oral glucose tolerance test; ACR, albumin:creatinine ratio; eGFR, estimated glomerular filtration rate. [file Image_1.jpeg]
